# Explaining the Timing of Natural Scene Understanding with a Computational Model of Perceptual Categorization

**DOI:** 10.1371/journal.pcbi.1004456

**Published:** 2015-09-03

**Authors:** Imri Sofer, Sébastien M. Crouzet, Thomas Serre

**Affiliations:** Cognitive, Linguistic & Psychological Sciences Department, Brown Institute for Brain Science, Brown University, Providence, Rhode Island, United States of America; Technische Universitat Chemnitz, GERMANY

## Abstract

Observers can rapidly perform a variety of visual tasks such as categorizing a scene as open, as outdoor, or as a beach. Although we know that different tasks are typically associated with systematic differences in behavioral responses, to date, little is known about the underlying mechanisms. Here, we implemented a single integrated paradigm that links perceptual processes with categorization processes. Using a large image database of natural scenes, we trained machine-learning classifiers to derive quantitative measures of task-specific perceptual discriminability based on the distance between individual images and different categorization boundaries. We showed that the resulting discriminability measure accurately predicts variations in behavioral responses across categorization tasks and stimulus sets. We further used the model to design an experiment, which challenged previous interpretations of the so-called “superordinate advantage.” Overall, our study suggests that observed differences in behavioral responses across rapid categorization tasks reflect natural variations in perceptual discriminability.

## Author Summary

The speed of sight has fascinated scientists and philosophers for centuries. In the blink of an eye, observers can rapidly and effortlessly perform a variety of categorization tasks such as categorizing a scene as open, as natural, or as a beach. The past decade of work has shown that there exist systematic differences in behavioral responses across different categorization tasks: For instance, participants appear to be faster and more accurate at categorizing a scene as outdoor (i.e., superordinate level) compared to categorizing a scene as a beach (i.e., basic level). Here, we describe a computational model combined with human psychophysics experiments, which help shed light on the underlying mechanisms. Using a large natural scene database, we trained machine learning algorithms for different categorization tasks and showed that it is possible to derive confidence measures that accurately predict variations in participants’ behavioral responses across categorization tasks and stimulus sets. Using the computational model to sample stimuli for a human experiment, we demonstrated that it is possible to reverse the superordinate advantage, rendering human observers superordinate categorization slower and less accurate than basic categorization—effectively challenging previous interpretations of the phenomenon. The study further offers a vivid example on how computational models can help summarize and organize existing experimental data as well as plan and interpret new experiments.

## Introduction

Categorization is perhaps one of our most critical visual functions as it allowed our ancestors to distinguish friend from foe and the edible from the inedible. Observers can rapidly extract meaning from brief presentations of complex visual scenes [1]—far exceeding the best existing engineered artificial systems [2].

Observers can reliably perform a variety of categorization tasks [3] such as categorizing a scene as open, as outdoor, or as a beach. However, it has also been shown that there exist systematic differences in participants’ behavioral responses across categorization tasks. In particular, categorizing a scene as open or navigable (i.e., attribute level) necessitates shorter presentation times than categorizing a scene as a lake or a beach (i.e., basic level, see [4]). (Note that our definition of basic-levelness follows the common usage in vision science (see [5–13]) and reflects a logical [14] rather than functional definition of the basic level.) Similarly, participants appear to be faster and more accurate when categorizing a scene as outdoor (i.e., superordinate level) compared to categorizing a scene at a basic level [7, 9, 11, 13]. A very recent study further suggests that subordinate scene categorization is less sensitive and slower than basic level categorization [15].

Beyond the categorization of natural scenes, there exist systematic differences in behavioral responses for object categories across taxonomic levels with observers’ subordinate-level categorization (e.g., pigeons vs. other birds) being slower and less accurate than basic-level categorization (e.g., birds vs. non-birds, see [5]. Similarly, basic-level categorization (e.g., birds vs. dogs) has been shown to be slower than superordinate categorization (e.g., animals vs. non-animals, see [16]). Participants tend to be faster and more accurate at categorizing faces at the superordinate level (i.e. categorizing faces vs. non-faces) compared with categorizing faces at the familiarity level (famous vs. non-famous, see [6]). However, for both familiar faces and other individually-known familiar objects, categorization at the subordinate level is faster than at the basic level [17]. Similarly, there exist systematic differences in behavioral responses for different social inference tasks [12]: For instance, categorization at the level of intentionality is faster than categorization at the level of belief and personality.

Such systematic behavioral differences across categorization tasks are often taken as suggestive evidence for an underlying hierarchical organization of categorization processes with some categorization tasks taking precedence over others [5–13], but see also [18–20]. Overall, the past decade of research on visual categorization has produced a significant and rapidly increasing amount of data and, while systematic differences across categorization tasks have been well-characterized to date, little is known about the underlying mechanisms.

In this study, we describe a computational model to account for variations in participants’ behavioral responses (both accuracy and reaction time) across tasks and stimuli for the rapid categorization of natural scenes. Previous work has proceeded along two seemingly parallel paths (see [21, 22] for discussions) with a nearly exclusive focus on modeling either visual representations (see [23] for review) or categorization and decision-making (see [24] for review). Here, we implemented a single integrated paradigm that links perception with categorization processes.

**Fig 1:**
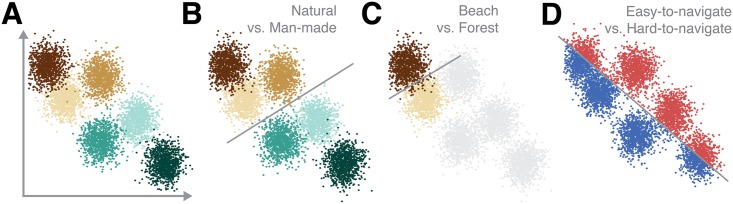
Principles of scene categorization. (A) Perceptual space: Visual features are first extracted from individual images, which can then be represented as datapoints in an *N*-dimensional space. (B–D) Categorization boundaries: The model assumes that different categorization tasks carve up the same perceptual space and correspond to different categorization boundaries (shown for hypothetical tasks: Superordinate level—‘natural’ vs. ‘man-made’ in (B), basic level—‘beach’ vs. ‘forest’ in (C) and scene attribute—‘easy’ vs. ‘hard’ to navigate in (D).

Formally, visual categorization corresponds to the process of associating visual stimuli **x**_*i* = 1…*m*_ to category labels *y*_*i* = 1…*m*_ to form (**x**_*i*_,*y*_*i*_) exemplar-label pairs. **x**_*i*_ may be parametrized by a feature vector in a *N*-dimensional perceptual space ${\text{x}}_{i}=(x_{i}^{1},\ldots x_{i}^{k},\ldots x_{i}^{N})$. Fig 1A illustrates such a feature space for an hypothetical population of *N* = 2 feature detectors (in practice, we expect *N* to be much larger). Learning to categorize visual stimuli requires learning a categorization boundary that best represents the relation between input images **x_*i*_** and their corresponding category labels *y*_*i*_. Once a categorization boundary had been learned, the classification of a stimulus depends on its position relative to the categorization boundary: One side of the categorization boundary will be associated with a target set of stimuli while the other side will be associated with the distractor set. An illustration for hypothetical decision boundaries corresponding to different taxonomic levels is shown in Fig 1B–1D. According to this computational framework, different categorization tasks correspond to different decision boundaries, which carve the same perceptual space, an idea that has motivated the development of most existing computational models of perceptual categorization (see [22] for review).

**Fig 2:**
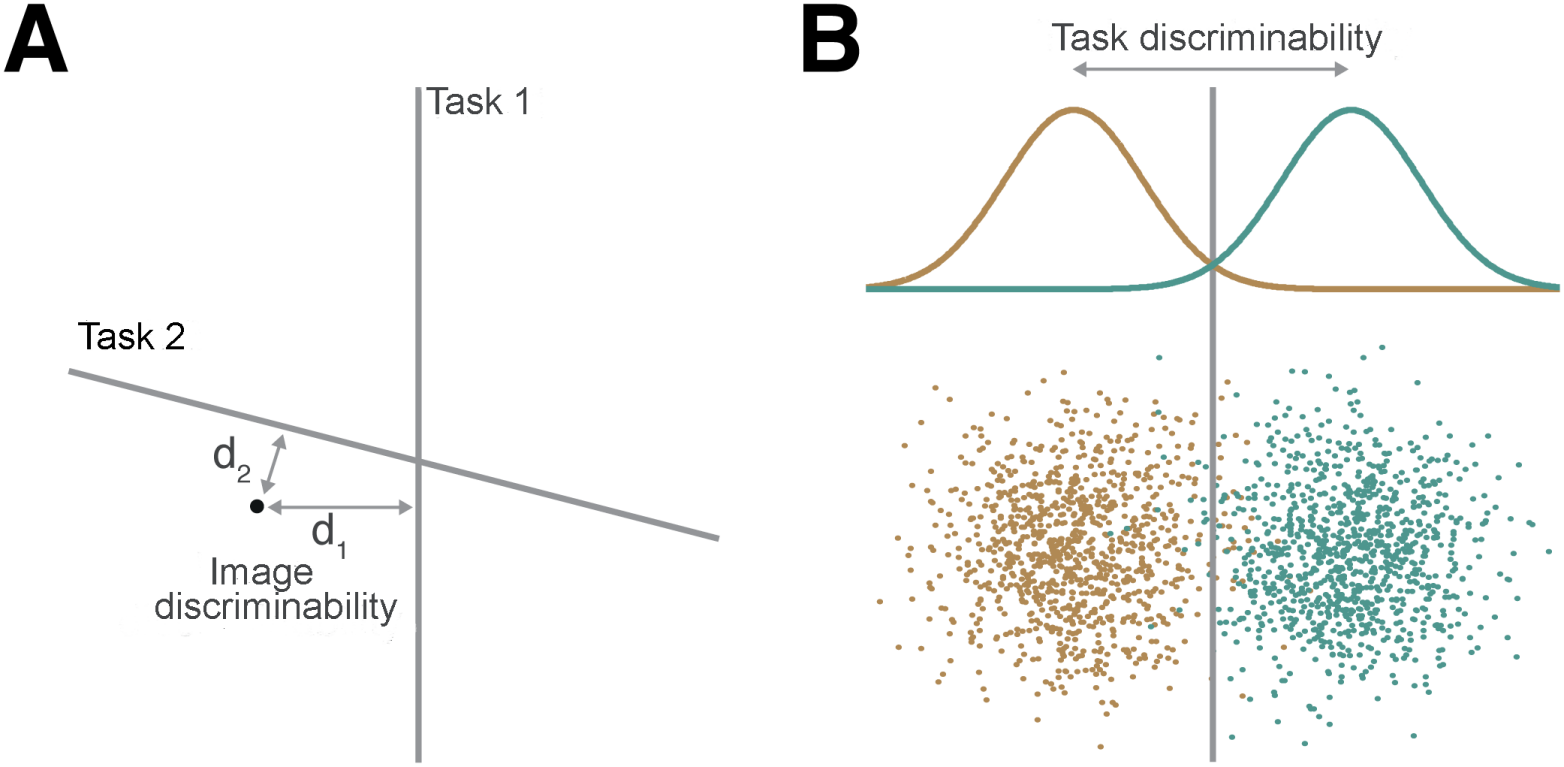
Computing discriminability values. (A) For an individual image and a specific categorization task (e.g., task 1), discriminability values are derived from the model by considering the distance *d*_1_ between the image and the categorization boundary associated with task 1. Here we tested the hypothesis that for a given stimulus and task, discriminability values drive participants’ average categorization accuracy and reaction times. (B) Discriminability values can also be computed for arbitrary sets of target (green) and distractor (brown) images. The normalized distance between these two distributions will determine how easy or difficult the task, as a whole, will be for human participants.

We used a rudimentary visual representation based on the “gist” algorithm [25] but other visual representations are possible (see [23] for review; see also Discussion). We further used a large image database [26] to train and test machine learning classifiers (regularized logistic regression) and estimate the decision boundaries associated with many different scene categorization tasks. A task-dependent measure of perceptual discriminability can then be derived for a particular categorization task by considering the distance between individual stimuli and the categorization boundary (Fig 2A). The basic intuition for this measure is that, for a particular categorization task, images that are closer to the categorization boundary will be harder to categorize than those that are further away leading to behavioral responses that are slower and less accurate. Furthermore, these values can be aggregated to yield estimates of accuracy for arbitrary sets of target and distractor stimuli (Fig 2B).

The goal of the present study was to test the hypothesis that the perceptual discriminability of individual stimuli for a particular task is one of the main factors driving behavioral responses. While this hypothesis is built-in for many categorization models (see [24] for review), it had so far only been tested with simple artificial stimuli where participants were trained to learn a new object category parametrized by two dimensions (e.g., [27], but see also [28] for alternative models.) However, this hypothesis has not yet been tested for well-learned, natural categories.

We first found that model-derived discriminability values predicted well behavioral responses for different categorization tasks as reported in two published studies [4, 11]. In addition, in experiment 1, we were further able to show that the model accurately predicted variations in accuracy and reaction time at the level of individual stimuli within the context of a scene categorization task. We then used the model to test the hypothesis that the so-called “superordinate advantage” [7, 9, 11, 13], whereby superordinate categorization is faster and more accurate than basic categorization, may reflect the greater perceptual discriminability of scenes at the superordinate vs. basic level. Consistent with this hypothesis, we first found that the model was consistent with the reported results of a published study on the superordinate advantage [9]. In experiment 2, we further showed that it is possible to use model-derived discriminability values to sample stimuli and to effectively reverse the superordinate advantage, making participants superordinate categorization slower and less accurate than basic categorization, thus offering a possible perceptual explanation of the phenomenon.

Overall, our results provide a computational-level explanation for the systematic variations in rapid categorization behavioral responses across taxonomic levels, suggesting that these differences may simply reflect natural variations in perceptual discriminability. Our study thus challenges several existing theories of visual processing and offers a vivid example of how computational models can help summarize existing data as well as plan and interpret novel experiments.

## Methods

### General methods

#### Ethics statement

The protocol was approved by Brown IRB [protocol #1002000135] and was carried out in accordance with the provisions of the World Medical Association Declaration of Helsinki. All participants reported having normal or corrected-to-normal vision and gave written informed consent.

#### Stimulus pre-processing

Images were converted to grayscale, cropped to a squared image, and then rescaled to 256×256 pixels. To minimize low-level brightness differences between targets and distractors, stimuli for each individual session were set to a constant mean brightness value (equal for all images in the corresponding session).

#### Computational model

The visual representation used here, called “gist” is relatively low-level [25, S1 Fig]. It was chosen for its simplicity in the absence of any strong evidence that a more complex visual representation would lead to significantly different model predictions (S2 Fig). Briefly, the image was first convolved with a bank of 32 Gabor filters (4 different scales in 8 orientations). The resulting convolution maps were then averaged separately in individual cells on a 4 × 4 grid covering the whole image. This yielded a 512-dimensional feature vector. Matlab source code for the “gist” is available online (see [25] for details).

Categorization boundaries were learned from natural scenes using a logistic regression classifier with L2 regularization. Software was implemented in Python using the scikit-learn [29] and the liblinear library [30]. Comparable levels of accuracy and qualitatively similar patterns of results were obtained with other types of classifiers (e.g., SVMs) as well as more complex kernels (see S1 Text). Categorization tasks were modeled as binary categorization tasks except in two of the comparisons with published results [9, 11], which were modeled using a one-vs-all multi-class classification approach.

Classifiers were trained and tested using cross-validation techniques whereby images were split into disjoint training and test sets multiple times at random (with replacement). The number of images sampled had to be varied across experiments because we tried to use the maximal number of samples available while creating datasets containing a balanced number of positive and negative samples. Except when noted, training and test data were split using an 80–20% training-test split. All hyper-parameters were optimized on the training set using a 5-fold cross-validation procedure.

Discriminability values were estimated by computing the average (test) classification error for each image in the dataset over multiple splits of the training/test data. This measure is simpler to compute than estimating the average distance between an image and the categorization boundary across all training/test data splits and, in practice, we found these two measures to agree closely.

Except when noted, the model accuracy was computed as the average rate of correct (test) classification over all random splits of the training/test data (*N* = 100, unless specified otherwise).

#### Apparatus and procedure

Participants sat in a dimly lit room. They were instructed to sit with their back leaning against the chair so as to maintain a viewing distance of approximately 75 cm to the CRT monitor (800 × 600 pixels, refresh rate of 140 Hz). Stimulus presentation was controlled using Matlab and the PsychToolbox [31] on a Mac Pro. Behavioral responses were collected using two handheld thumb button switches connected to a response time box [32].

**Fig 3:**
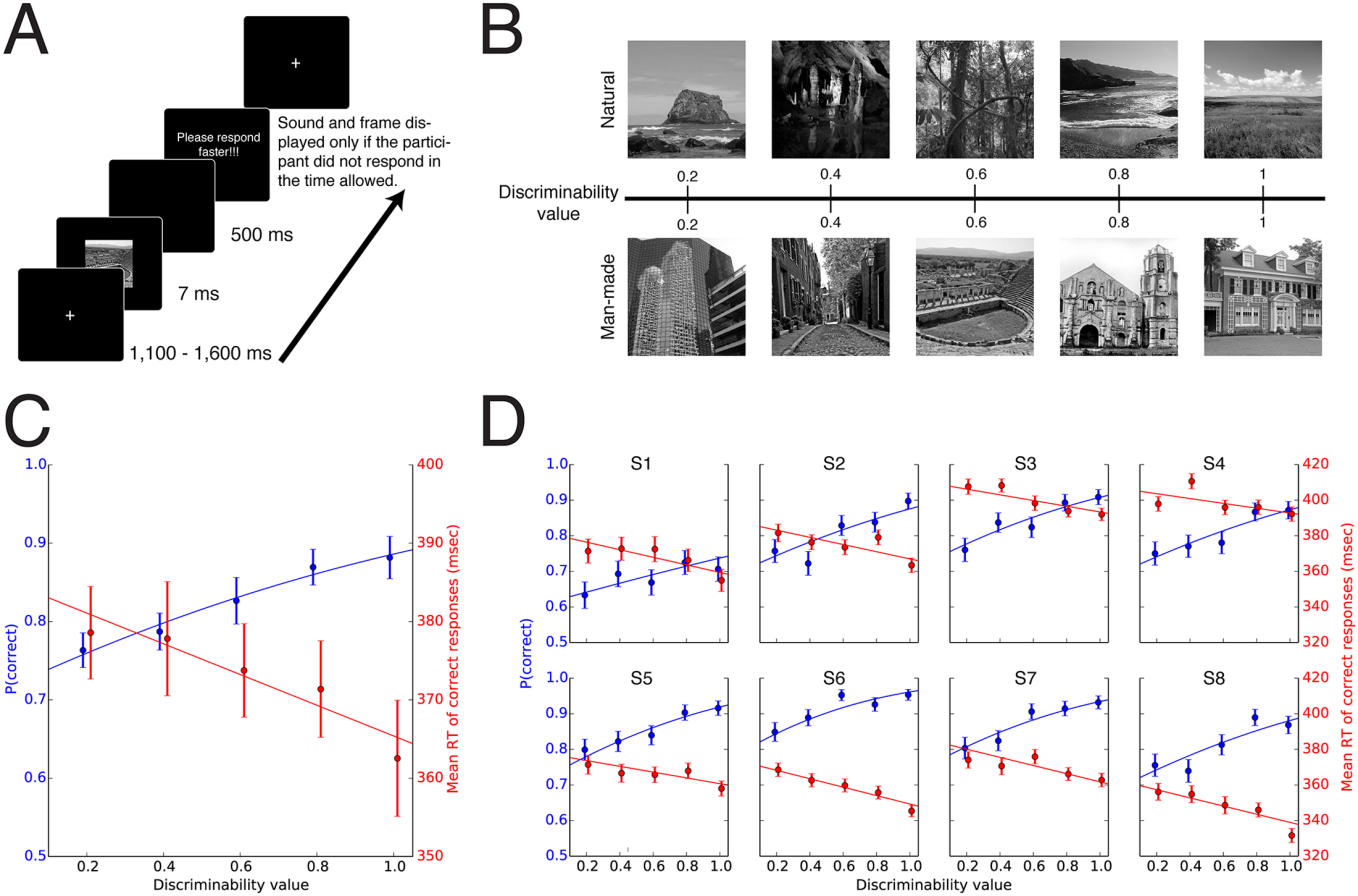
Experiment 1: Model discriminability values predicted participants mean accuracy and RTs. (A). Overview of the experimental design: Each trial began with a fixation cross followed by the subsequent brief presentation of an image (7 ms). Participants were required to respond within 500 ms. (B) Representative scenes sampled at five distinct discriminability value levels for a natural vs. man-made categorization task. Note that the original stimuli used could not be shown because of copyright and were replaced instead by visually similar images found on Flickr under the creative common. (C) Average results across all participants: Accuracy (percentage of correct responses, blue) and mean reaction time (RT) for correct responses (red) as a function of discriminability values as predicted by the model. Curves correspond to a GLMM fit and error bars to the standard deviation of the mean. (D) Results for individual participants.

On each trial, the experiment ran as follows: On a black background (1) a fixation cross appeared for a variable time (1,100–1,600 ms); (2) a stimulus (10° × 10°) was presented for a single frame (7 ms). The order of image presentations was randomized. Participants were instructed to answer as fast and as accurately as possible by pressing the button in their strong hand if they saw a target, and the other button if they saw a distractor. Participants were forced to respond within 500 ms (a sound was played and a message displayed in the absence of a response past the response deadline). At the end of each block, participants received feedback about their accuracy. An illustration of the experimental paradigm is shown in Fig 3A.

#### Sample size and stopping criterion

Here, we applied a Bayesian analysis of results (see below). Thus, there was no need to predetermine a stopping rule or sample size, as the analysis does not depend on the researchers’ intentions [33].

### Predicting behavioral categorization based on discriminability: Existing literature and experiment 1

#### Model initial validation

We initially validated the model by postdicting behavioral categorization results reported in [4, 11]. Because of the relatively small size of the image dataset, the proportion of training images relative to test images had to be increased to reproduce the results described in [4] (96/4% training/test split). Because of the nature of the task in [11], the accuracy measure *acc*_*i*,*j*_ for discriminating between category *i* and *j* was computed as 1 minus the fraction of images from category *i* classified as category *j* and vice-versa.

#### Using the model to sample stimuli

The model was trained to discriminate between ‘natural’ and ‘man-made’ scenes using the SUN database [26], which is currently the largest available scene database with nearly 400 basic level categories for a total of approximately 100,000 images. We selected the following man-made categories: ‘skyscraper,’ ‘highway,’ ‘street,’ ‘tower,’ ‘alley,’ ‘apartment building,’ and ‘amphitheater’ and the following natural categories ‘beach,’ ‘desert,’ ‘cultivated field,’ ‘coral reef,’ ‘iceberg,’ ‘forest’ (by combining ‘broad leaf tree,’ ‘needle leaf tree’ and ‘rain forest’), ‘mountain’ (by combining ‘mountain,’ ‘snowy mountain,’ ‘coast,’ and ‘cliff’).

Decision values were estimated for each individual image using the procedure described in *General methods*. We binned stimuli according to their associated discriminability values (5 bins for each superordinate category: 0.2, 0.4, 0.6, 0.8, and 1.0) and sampled 96 images for each bin. Each sampled image was inspected manually. If the category label for the image was found to be ambiguous (e.g., a house in a prairie may yield some ambiguity in the corresponding class label because a house is man-made but a prairie is natural), a stimulus was re-sampled from the same bin. For each level, the resulting mean discriminability values for the chosen images was computed to ensure that it remained close to the target discriminability values.

#### Experimental paradigm

A total of 8 participants completed the experiment (6 males, 2 females; mean age 21 years, range 20–26; all right-handed). All participants reported having normal or corrected-to-normal vision and gave written informed consent.

The experiment followed the experimental design described in *General methods*. We used a within-subject factorial design: 2 categories (man-made vs natural) × 5 discriminability values (0.2, 0.4, 0.6, 0.8, and 1.0) derived from the model. Participants first viewed 20 natural and 20 man-made images randomly selected from the target and distractor image sets (4 from each discriminability value). Participants subsequently completed 16 blocks total. In each block, 6 images from each condition were presented in a random order leading to 60 images in each block and 960 trials in total.

#### Analysis of results

Signal detection theory can be formulated as a special instance of a generalized linear model to estimate experimental effects on participants [34]. It can also be extended to the population level using generalized linear mixed effect models [35], thus providing a very powerful and efficient estimation technique. In addition, mixed effect models are equivalent to Bayesian hierarchical models with an uninformative prior [36]. Therefore, this analysis did not suffer from the common drawbacks associated with null hypothesis significance testing [33].

The response *y* of each participant was modeled as:

$$y=probit^{-1}\left(\beta_{bias}+\beta_{sens}x_{sens}+\beta_{slope}x_{slope}\right),$$

where *probit* ^−1^ denotes the cumulative distribution function of the standard normal distribution, *β*_*bias*_ the response bias of the participant, and *β*_*sens*_ the participant’s sensitivity for categorizing ‘natural’ vs. ‘man-made’ images for a middle discriminability target value (discriminability value = 0.6). *β*_*slope*_ corresponds to the change in sensitivity associated with a change in discriminability value, and it is the parameter of interest for this analysis. For each trial, the participant’s response was set to 1 for ‘man-made’ responses and, and 0 for ‘natural’ responses. *x*_*sens*_ was set to 0.5 for man-made trials, and −0.5 for natural trials. *x*_*slope*_, which codes for the discriminability value, was set to (−2,1,0,1,2) for natural images with corresponding discriminability values (1,0.8,0.6,0.4,0.2). For the man-made category, a reversed coding scheme was used.

The RT at trial *i* for each subject was modeled in a similar manner:

$$RT=\beta_{0}+\beta_{bias}x_{bias}+\beta_{slope}x_{slope},$$

with *x*_*slope*_ coded as (−2,1,0,1,2) for images with associated discriminability values (1,0.8,0.6,0.4,0.2). P-values and confidence intervals for all experiments and model analyses were estimated using *n* = 10,000 Monte Carlo samples. P-values referred to a two-tailed test.

### Using the model to reverse the superordinate advantage: Existing literature and experiment 2

#### Model initial validation

We initially validated the model by postdicting behavioral categorization results reported in [9] (experiment 1 and 2), using the procedure described in General Methods. The model was trained and tested using the 8-scene database [4] (which constitutes a superset of the manually sampled subset used in [9]).

#### Using the model to sample stimuli

For this experiment, we used the computational model to create sets of natural and man-made stimuli. First, sets of stimuli were obtained by considering combinations of 3 basic categories from a larger set of natural (‘beach,’ ‘desert,’ ‘cultivated field,’ ‘coral reef,’ and ‘iceberg’) and man-made (‘skyscraper,’ ‘highway,’ ‘street,’ ‘tower,’ ‘alley,’ ‘apartment building,’ and ‘amphitheater’) categories. The computational model was then tested on all possible combinations of natural and man-made sets for categorization at the superordinate level (man-made / target vs. natural / distractor categorization) and at the basic level (forest / target vs. natural / distractor categorization) as done in [4]. The same set of natural stimuli was used as distractor for both categorization tasks.

We subsequently chose two sets of natural and two sets of man-made stimuli to create two experimental conditions: a *superordinate advantage* condition for which the model predicted high perceptual discriminability for superordinate-level categorization but low discriminability for basic-level categorization and a *basic advantage* condition for which the model predicted the opposite trend (low perceptual discriminability for superordinate-level categorization and high discriminability for basic-level categorization). This yielded the following category combinations for the superordinate advantage condition: ‘beach,’ ‘cultivated field,’ and ‘coral reef’ for natural categories and ‘alley,’ ‘street,’ and ‘skyscraper’ for man-made stimuli and the following category combinations for the basic advantage condition: ‘beach,’ ‘iceberg,’ and ‘desert’ for the natural categories while the man-made categories were ‘alley,’ ‘amphitheater,’ and ‘highway.’

For each experimental condition, 168 images were randomly sampled from both target and distractor categories. All sampled images were inspected visually and images for which the associated class label was deemed ambiguous were replaced by a randomly sampled image. To generate predictions for individual tasks, we re-trained the classifiers using the cross-validation procedure described in *General methods*.

#### Experimental paradigm

A total of 24 participants completed the experiment (8 males, 16 females; mean age 24 years, range 18–25; all right-handed). All participants reported having normal or corrected-to-normal vision and gave written informed consent.

The experiment started with a practice block for an unrelated rapid categorization task (animal vs. non-animal) to familiarize participants with the experimental paradigm. The experiment began after participants correctly categorized 75% of the images in a single practice block. In addition, participants were allowed to browse through the stimulus set used in the session before the main experiment to familiarize themselves with the task.

We used a mixed design: 2 conditions (superordinate advantage and basic advantage) × 3 target categories (forest, mountain, and man-made). Half of the participants were assigned to the superordinate advantage condition and half to the basic advantage condition. Three tasks were tested: one superordinate (man-made vs. natural) and two basic categorization tasks (forest vs. natural and mountain vs. natural). Each participant completed 18 blocks (6 blocks for each task, 56 stimuli per block, 336 stimuli per task for a total of 1,008 trials). The order of the blocks was counterbalanced across participants. Each target image appeared only once for the entire experiment while each distractor appeared 3 times (once for each task). In each block, targets and distractors appeared with an equal probability. The target category was indicated at the beginning of each block with a written instruction on the screen together with 16 random exemplar images (8 targets and 8 distractors).

#### Analysis of results

The original experiment included three tasks: 1 superordinate and 2 basic-level (forest and mountain) categorization tasks. The superordinate and the forest categorization tasks were the main factors tested in the experiment, and the mountain task was introduced to collect additional data. However, we observed a pervasive influence of a speed-accuracy tradeoff (SAT) for the mountain task: Participants appeared to be using a different SAT criterion (they were either more accurate and slower or less accurate and faster) and behavioral responses for this task could not simply be compared to behavioral responses for the other two tasks. This result did not conflict with our main hypothesis that the superordinate advantage can be reversed and the task was simply excluded from further analysis.

The behavioral response *y* of each participant was modeled as:

$$y=probit^{-1}\left(\beta_{bias}+\beta_{sens}x_{sens}+\beta_{cont}x_{cont}\right),$$

where *probit* ^−1^, *β*_*bias*_ and *β*_*sens*_ were defined as in experiment 1, and *β*_*cont*_ corresponded to the change in sensitivity between the superordinate and the basic-level categorization task. For this analysis, *β*_*cont*_ was the key parameter of interest. This formulation is similar to a two-factor anova, where *β*_*bias*_ represents the first main effect, *β*_*sens*_ the second main effect, and *β*_*cont*_ the interaction.

As for experiment 1, for each trial, *y* was set to 1 if the participant pressed the target button, and 0 otherwise (non response trials were omitted). *x*_*sens*_ was set to 0.5 for target trials and −0.5 for distractor trials. *x*_*cont*_ was set to 0.25 for superordinate/target trials and for basic/distractor trials, and it was set to −0.25 for superordinate/distractor trials and for basic/target trials. All parameters were set as random effects to allow them to vary for each individual participant. The same model was fitted to each condition separately, and from each, one can derive the sensitivity for the two tasks in that condition:

$$\text{Basic:}\;\beta_{sens}-0.5\beta_{cont}.$$

$$\text{Superordinate:}\;\beta_{sens}+0.5\beta_{cont}.$$

The model used for RTs was similar, albeit simpler, since we only used correct trials. For each individual trial and subject, the RT was modeled as:

$$RT=\beta_{0}+\beta_{bias}x_{bias}+\beta_{cont}x_{cont},$$

where *β*_0_ denotes the mean RT, *β*_*bias*_ the response bias, and *β*_*cont*_ the change in RT between the two tasks. *x*_*bias*_ was set to 0.5 for target trials −0.5 for distractor trials. *x*_*cont*_ was set to 0.5 for the superordinate-level categorization task and to -0.5 for the basic-level categorization task. Monte-Carlo samples (*n* = 10,000) were used to estimate p-values and confidence intervals for all experiments and analyses. P-values refer to two-tailed test.

## Results

### Predicting behavioral categorization based on discriminability: Existing literature and experiment 1

#### Model initial validation

As an initial validation of the model, we considered two representative rapid scene categorization studies [4, 11] to compare the model’s predicted perceptual discriminability for different categorization tasks (across taxonomic levels) against human behavioral responses. For both studies, we trained the computational model using the stimuli set from the original experiments, and assessed the model’s discriminability for the same tasks. We then compared the model discriminability scores against human behavioral responses (as reported in the original studies).

**Fig 4:**
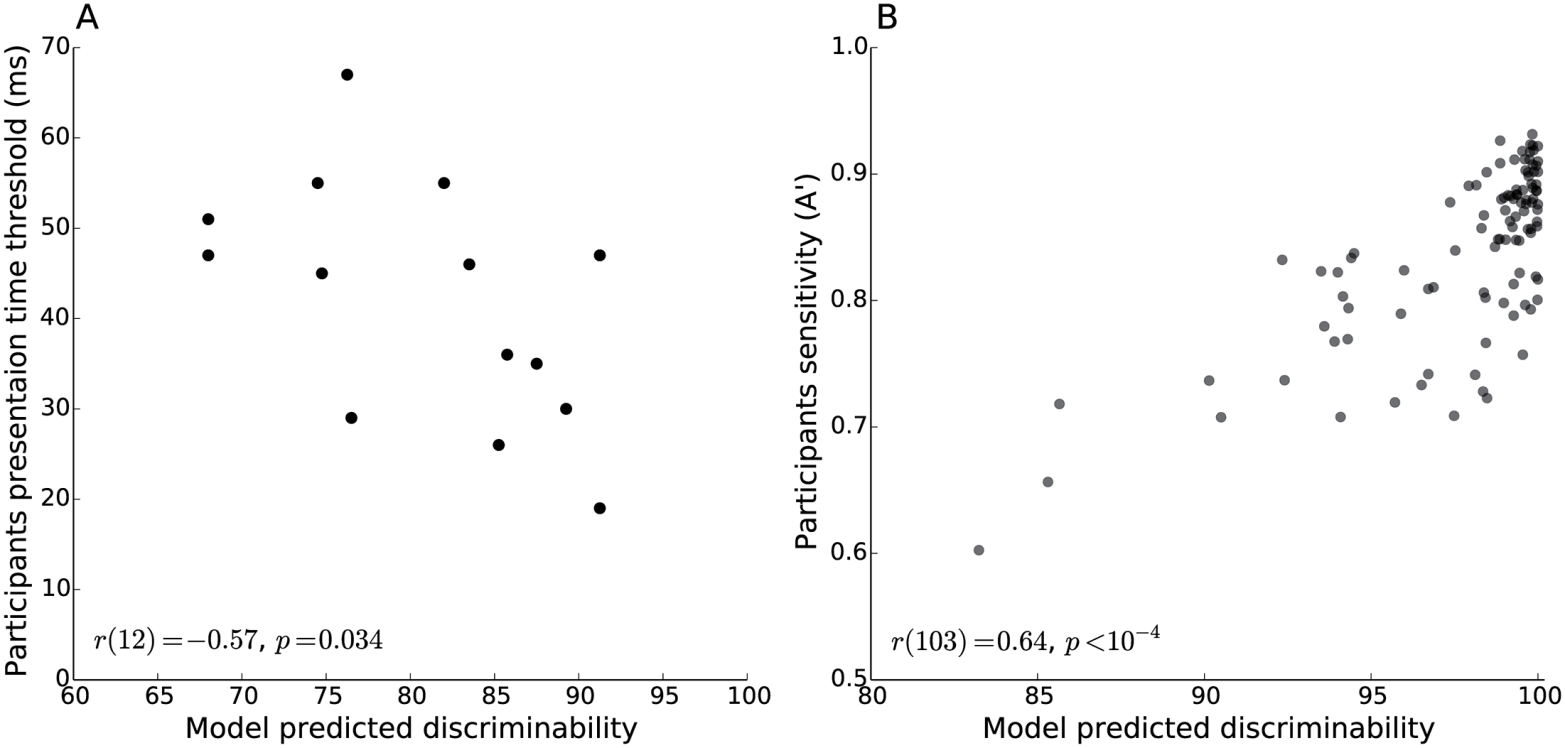
Comparison between the model predicted task discriminability against human accuracy on multiple scene categorization tasks based on two representative studies. (A) Negative correlation between the model predicted task discriminability and participants’ presentation time threshold [4]. (B) Positive correlation between the model predicted task discriminability and participants sensitivity [11] (a small jitter was added to the display in (B) to improve visualization).

In [4], the authors used a staircase procedure to estimate the presentation duration needed for participants to reach a fixed level of accuracy for fourteen distinct scene categorization tasks [4]. These tasks were based on either scene attributes (concealment, depth, naturalness, navigability, openness, temperature, and transience) or basic level category membership (desert, field, forest, lake, mountain, ocean, and river). We took participants’ presentation time thresholds as reported in [4] and compared them to the model-predicted perceptual discriminability. As expected we found them to be negatively correlated (Spearman correlation; *r*(12) = −0.57,*p* = 0.03; Fig 4A).

In [11], the authors looked at the rate at which participants classify two masked images, which belong to different categories, as belonging to the same category. This rate was used to define the perceptual similarity between any two categories. The authors tested all possible pairs of 15 categories, which resulted in 105 pairs of categories overall. We correlated the human sensitivity scores reported in [11] for individual tasks against the discriminability predicted by the model for the same tasks (Spearman correlation; *r*(103) = 0.64,*p* < 10^−4^; Fig 4B).

Overall, discriminability values derived from the computational model appeared sufficient to account for observed participants’ variations in behavioral responses for a relatively large and disparate number of tasks across experiments. Beyond this initial model validation, we will next show that it is possible to use the model to sample stimulus sets in order to systematically manipulate participants’ behavioral responses.

#### Experiment 1

We assessed the accuracy and RTs from human participants using a rapid man-made vs. natural scene categorization paradigm. Images were sampled using discriminability values derived from the model. Sample images for each level of discriminability are shown in Fig 3B. On average, participants answered correctly on 83.0% of the trials (±2.4%). Trials for which participants failed to answer before the deadline were excluded from further analysis (5% of the total number of trials). The mean RT for correct responses was 372 ms (±7 ms), and is comparable to previously published results [7].

The model predicted a monotonic increase in accuracy and corresponding monotonic decrease in reaction time as a function of the stimulus discriminability values on either side of the categorization boundary. We thus fitted one generalized linear mixed effect model (GLMM) to behavioral responses to estimate the change in the rate of correct responses as a function of discriminability values and one separate GLMM to RTs (Methods). Decision values were found to have a significant effect at the group level for both accuracy (*β*_*slope*_ = 0.14, 95% confidence interval *CI* = [0.10,0.17], *p* < 10^−4^) and RT (*β*_*slope*_ = 3.92,*CI* = [2.85,5.02],*p* < 10^−4^). Results are shown in Fig 3C. These group-level results also held for individual participants as shown in Fig 3D (*p* < 10^−3^ for all participants).

These results validate the model key hypothesis that, for a given categorization task, variations in behavioral responses across stimuli are accounted for by the predicted stimulus’ perceptual discriminability for that particular task. Could natural variations in task discriminability thus also account for systematic variations in behavioral responses found across categorization tasks—including differences reported across taxonomic levels as exemplified by the “superordinate advantage”?

### Using the model to reverse the superordinate advantage: Existing literature and experiment 2

#### Model initial validation

**Fig 5:**
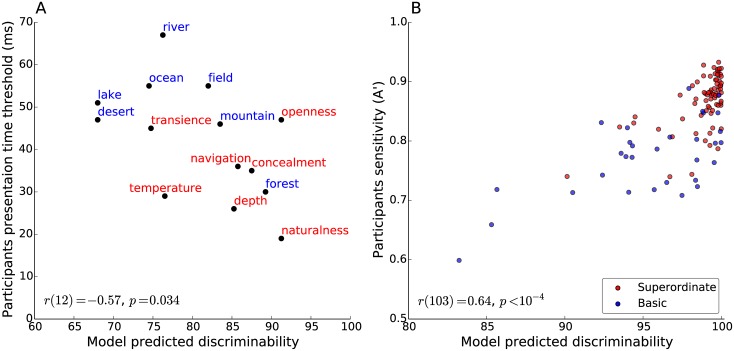
Re-drawing of Fig 4 with labels indicating the taxonomic level of individual categorization tasks. (A) Attribute-level categories are labeled in blue and basic-level categories in red. (B) Basic-level categories are labeled in blue and superordinate-level categories in red.

A re-drawing of Fig 4 with the addition of color labels to indicate the taxonomic levels of the different tasks used in [4, 11] makes it clear that behavioral differences between taxonomic levels (attribute vs. basic level in Fig 5A or basic vs. superordinate level in Fig 5B) can be also explained by differences in perceptual discriminability. That is, the perceptual discriminability, as postdicted by the model for the attribute and superordinate categorization tasks used in [4] and [11] respectively, tend to be higher than for the corresponding basic categorization tasks. In addition, the model correctly postdicted the presentation threshold for the ‘forest’ (basic) category (which appeared to be faster than most attributes) or the ‘transience’ (attribute) category (which was comparable in speed to several basic level categories)—two categories that would be considered as outliers under a level-of-categorization interpretation.

These initial results suggest that the superordinate advantage could simply reflect natural variations in discriminability between different target and distractor sets. To explicitly test this hypothesis, we used data by [9] and found that the model postdicted a higher perceptual discriminability for their superordinate-level vs. basic-level categorization tasks and a lower discriminability for categorization between two basic categories that belong to the same superordinate class (e.g., both natural) compared to categorization between two basic categories that belong to different superordinate classes.

In [9], participants were tested on different categorization tasks using a backwards masking paradigm. In a first experiment, one group of participants performed a superordinate-level categorization task while another group performed a basic level categorization task. Consistent with participants’ behavioral responses [9], the model correctly postdicted a higher perceptual discriminability for superordinate vs. basic categorization as measured by the difference in sensitivity (*A*′) between superordinate and basic categorization (Human: *M* = 0.05±0.02; Model: *M* = 0.03±0.01) (see [9] for details). In a second experiment, participants had to discriminate between two basic categories that either belonged to the same or different superordinate categories. Again, consistent with participants’ behavioral responses [9], the model correctly postdicted a lower discriminability for categorization between two basic categories that belong to the same superordinate class (e.g., both natural) compared to categorization between two basic categories that belong to different superordinate classes. This effect was measured using the difference in sensitivity between the “same” task and the “different” task (Human: *M* = 0.14±0.06; Model: *M* = 0.05±0.01). Next, we demonstrate the contribution of perceptual discriminability to the superordinate advantage more directly by showing that it was possible to sample stimuli based on model-derived discriminability values to reverse the superordinate advantage—rendering a superordinate categorization task harder for human participants compared to a basic level categorization task.

#### Experiment 2

**Fig 6:**
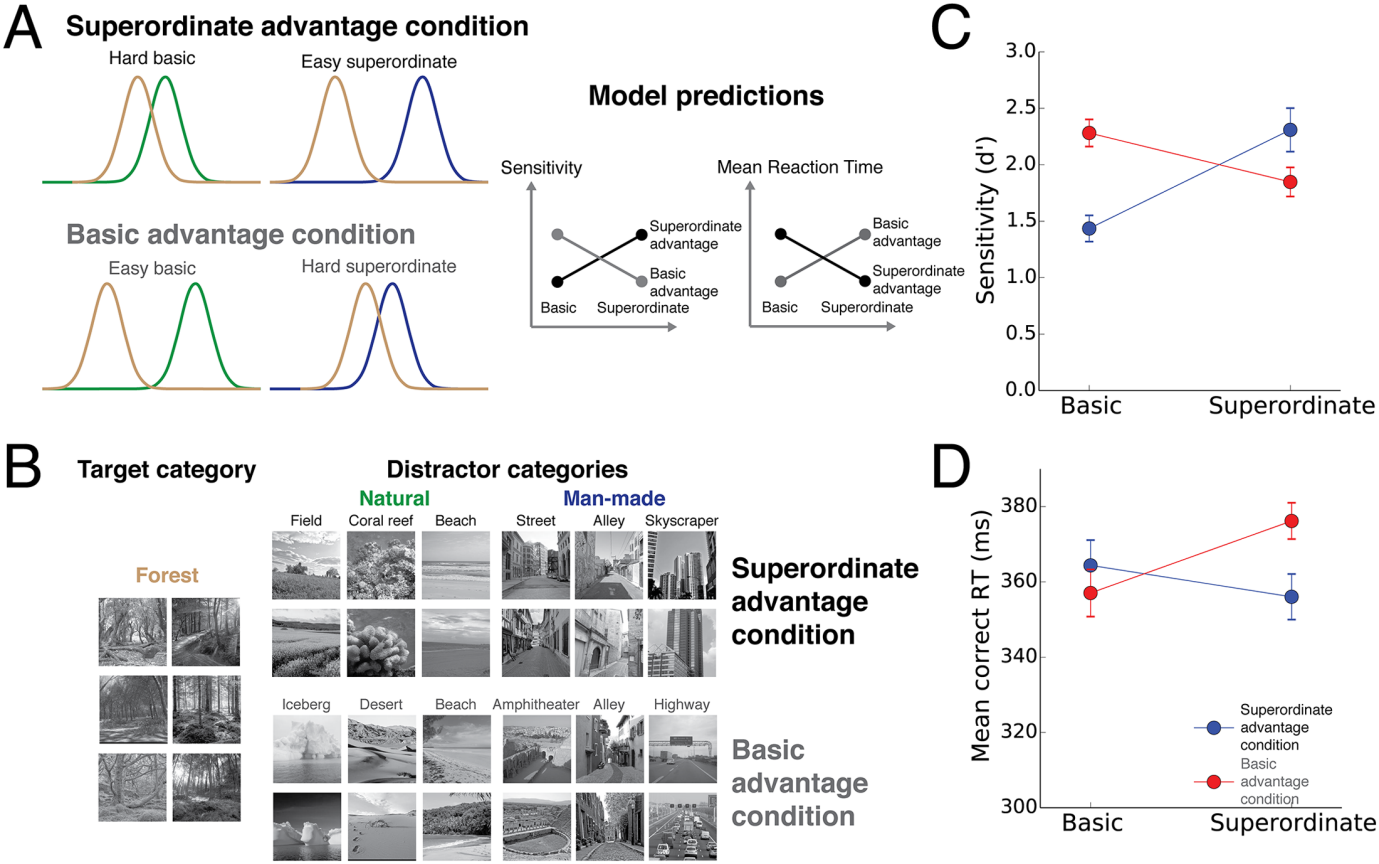
Experiment 2: Reversing the superordinate advantage. (A). Model discriminability values were used to sample stimulus sets to yield a high discriminability for superordinate categorization and a low discriminability for basic categorization to try to replicate the superordinate advantage (“superordinate advantage” condition) as well as a low discriminability for superordinate categorization and a high discriminability for basic categorization to try to reverse the superordinate advantage (“basic advantage” condition). (B) Representative images used in the experiment. Note that the original stimuli could not be shown because of copyright issues. Instead, shown are visually similar images from Flickr with a Creative Common licence. (C) Experimental results: The model correctly predicted higher accuracy and lower mean RTs for the superordinate vs. basic categorization task in the superordinate advantage condition and the opposite trend in the basic advantage condition.

We sampled stimuli from the SUN database using model discriminability values to yield either high discriminability for superordinate categorization but low discriminability for basic categorization to try to replicate the superordinate advantage (“superordinate advantage” condition) and a low discriminability for superordinate categorization and a high discriminability for basic categorization to try to reverse the superordinate advantage (“basic advantage” condition; Fig 6A). In each condition, participants had to perform both a superordinate (man-made vs. natural) and a basic categorization task (forest vs. natural). The only difference between the two conditions was the set of target and distractor stimuli used, which were both sampled from the SUN image dataset as in Experiment 1 (Fig 6B).

**Fig 7:**
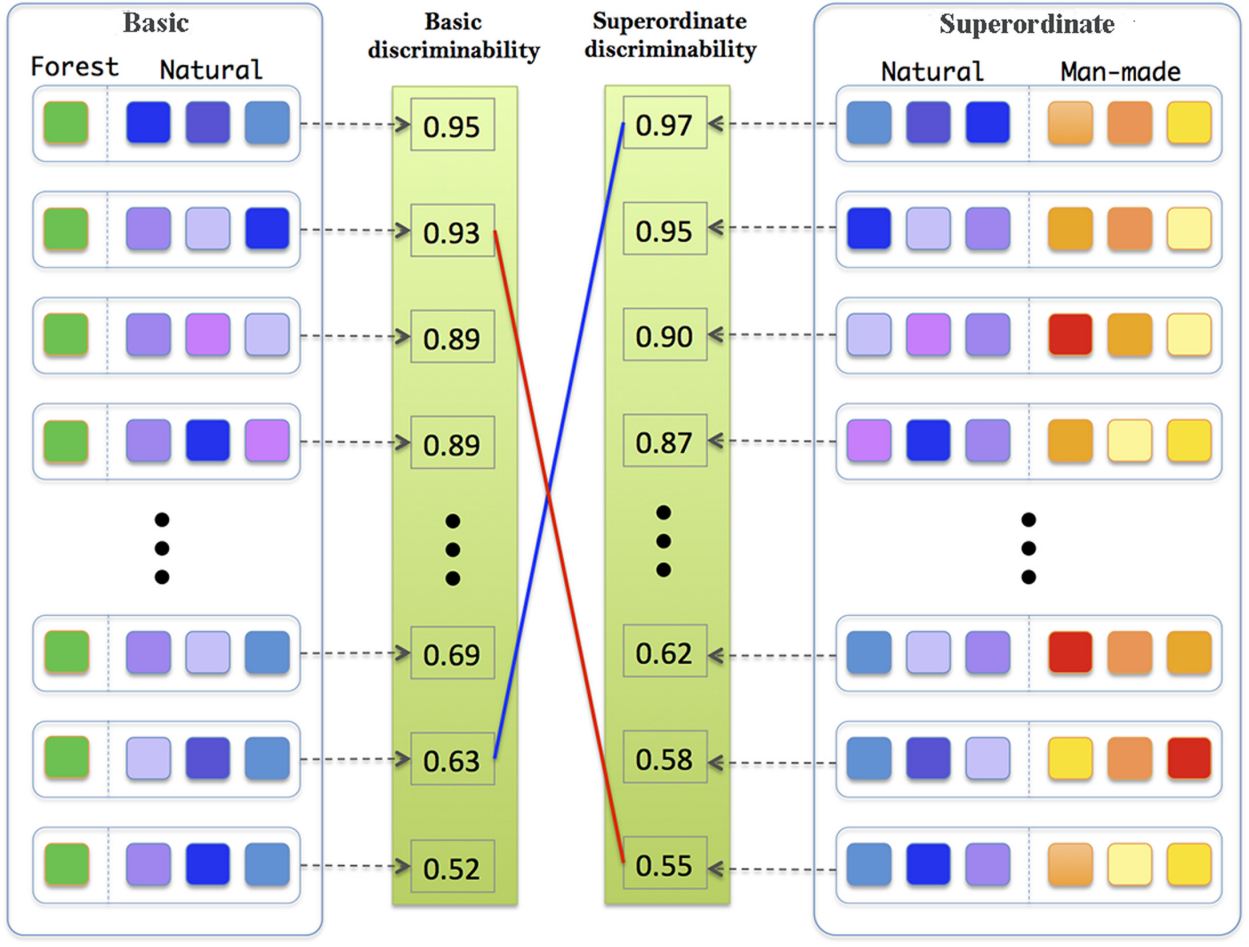
Reversal category picking framework. We created many different image datasets to train and test the model on both a basic level categorization task (forest vs. natural stimuli) and a superordinate categorization task (man-made vs. natural stimuli). This was done by considering all possible combinations of 3 basic categories from a larger set of natural categories and all possible combinations of 3 basic categories from a larger set of man-made categories. We computed discriminability values for all the corresponding categorization tasks and chose natural and man-made combination sets of stimuli to create 2 experimental conditions: (1) A *superordinate advantage* condition for which the model predicted high perceptual discriminability for superordinate-level categorization but low discriminability for basic-level categorization (blue line). The combination set included ‘beach,’ ‘cultivated field,’ and ‘coral reef’ for natural categories and ‘alley,’ ‘street,’ and ‘skyscraper’ for man-made stimuli. (2) A *basic advantage* condition for which the model predicted the opposite trend (low perceptual discriminability for superordinate-level categorization and high discriminability for basic-level categorization, red line). The combination set included: ‘beach,’ ‘iceberg,’ and ‘desert’ for the natural category while the man-made category included ‘alley,’ ‘amphitheater,’ and ‘highway.’

Both the man-made and the natural superordinate categories consisted of images from three basic categories (Fig 6B). However, across conditions, different basic categories were chosen. This was done by running a large number of model simulations. Overall, for the superordinate task, we used all possible combinations of 3 man-made basic categories against all possible combinations of 3 natural basic categories (Fig 7). We simulated the basic categorization task by categorizing the forest category against all combinations of three natural categories. For each condition, we then obtained categories that maximized the difference between the superordinate and the basic tasks (see Methods for details).

As in Experiment 1, we used a GLMM to analyze participants’ sensitivity and mean RT for correct responses (Fig 6C). In the *superordinate advantage* condition, the average sensitivity was 2.31 (±0.19) for the superordinate task and 1.44 (±0.12) for the basic task. The within-subject difference in sensitivity was large and significant (*β*_*cont*_ = 0.87,*CI* = [0.64,1.11], *p* < 10^−4^). Mean RTs were 356 ms (±6 ms) for the superordinate task and 364 ms (±6 ms) for the basic task. The within-subject difference in mean RT was significant as well (*β*_*cont*_ = 8.36,*CI* = [0.11,16.63], *p* = 0.050).

The opposite pattern was observed in the *basic advantage* condition. The average sensitivity was 1.85 (±0.13) in the superordinate task, and 2.28 (±0.12) in the basic task. The within-subject difference in sensitivity was smaller than the other group but still highly significant (*β*_*cont*_ = 0.43,*CI* = [0.24,0.63], *p* < 10^−4^). The mean RT was 376 ms (±5 ms) for the superordinate task, and 357 ms (±6 ms) for the basic task. The within-subject difference in mean RT was again large and significant (*β*_*cont*_ = 19.14,*CI* = [12.01,26.5], *p* < 10^−4^).

## Discussion

We have described an integrated paradigm that links perceptual processes with categorization processes. We used a large natural scene database to train and test machine learning classifiers in order to derive task-dependent perceptual discriminability values for individual images based on their distance to different categorization boundaries. We showed that the resulting model is consistent with a host of published results [4, 9, 11]. In addition, we also designed two experiments to demonstrate that it is possible to use the model to sample stimuli in order to manipulate participants’ behavioral responses (both accuracy and reaction times).

In experiment 1, we showed that sampling stimuli with increasing discriminability values (i.e. with increasing distance to the category boundary) yields behavioral responses that are increasingly fast and accurate. This suggests that the perceptual discriminability of individual stimuli for a particular task is one of the main factors driving behavioral responses.

A few recent studies have hinted at the contribution of perceptual discriminability to categorization using isolated objects [37], objects in clutter [23] and scenes [38, 39]. It has been shown that the perceptual dissimilarity between categories directly affects the speed of superordinate-level vs. basic-level categorization in pigeons [40]. Early work on scene and face processing already hinted at this contribution by showing, for instance, that the stimulus content across spatial scales affects scene categorization performance [41]. Subsequent work has also shown that the manipulation of the phase and amplitude spectra of an image affects behavioral responses during scene superordinate categorization [42, 43]. More recently, it has been shown that a low-level perceptual similarity measure based on stimulus contrast predicts the ease of categorization judgments for both artificial stimuli [44] and natural scenes [45]. Our study further demonstrates that it is possible to use modern machine learning tools and computer vision databases to predict human behavioral responses for many categorization tasks across taxonomic levels.

In experiment 2, we further showed that it is possible to use the model to sample stimuli in order to reverse the “superordinate advantage” rendering participants’ superordinate categorization arbitrarily slower and less accurate than basic categorization. Previous work has shown that it is possible to manipulate level-of-categorization effects by controlling the similarity between face stimuli [46] and the typicality of objects [47]. Here, we used the model to sample stimuli based on computed discriminability values, possibly making a superordinate categorization task harder compared to a basic level categorization task simply by sampling the right stimuli.

Our results suggest that the superordinate advantage is at least in part driven by the perceptual discriminability of target and distractor stimulus sets. Simply put, superordinate-level categorization tasks tend to be easier than basic-level categorization tasks leading to observers’ behavioral responses that are faster and more accurate. This is consistent with the somewhat higher accuracy of both connectionist models [48] and modern computer vision systems for categorization at the superordinate vs. basic level [49] and is consistent with the fact that children learn to categorize natural object categories at the superordinate level first [48, 50].

Our results are consistent with the differentiation theory [51] and the Parallel Distributed Processing (PDP) theory [52] in that level-of-categorization effects as reported in multiple studies [5–7, 9, 11–13] arise, not because of privileged processing at particular taxonomic levels, but because of differences in perceptual discriminability across tasks. In addition, this perceptual explanation rules out an interpretation of level-of-categorization effects based on the “global-to-specific” theory of categorization, whereby categorization at more global (coarser) categorization stages need to be completed before categorization at more specific (finer) levels can begin. Hence, one would expect a basic advantage over subordinate categorization (e.g., detection preceding identification [53]) as well as a superordinate and attribute advantage over basic and subordinate categorizations [4, 5, 11]. Our results demonstrate that observed differences in timing across categorization tasks do not necessarily reflect the fact that some categorization tasks take precedence over others (see also [19, 20]).

While our results point to perceptual discriminability as playing a fundamental role in level-of-categorization effects, additional memory-related factors such as typicality are likely to affect rapid categorization. More generally, a complete model should also take into account known semantic contributions to visual categorization. One proposal is that mental representations of categories across taxonomic levels occupy nodes in a semantic network [54]. The rapid perceptual categorization mechanisms studied here may determine which nodes get activated first before activation spreads to other nodes enabling the slower retrieval of information at other levels of categorization [52].

The present study also has implications for models of category learning and models on the development of visual expertise. It is known that experts can override the supremacy of one level of categorization found in novices with their own level of expertise (e.g. the subordinate level becomes faster for bird experts that are over-trained at the subordinate level, the basic-level becomes faster for Chinese character experts that are over-trained in discriminating characters at the basic level (irrespective of font and writing style, see [22] for review). One simple explanation consistent with our results is that practice for a task leads to long-term perceptual learning that increases the discriminability between targets and distractors, making participants faster and more accurate.

Despite its ability to account for behavioral responses, the proposed model remains relatively simple. We used a rudimentary visual representation based on the “gist” algorithm [25] and off-the-shelf machine learning classifiers (see [39] for a similar model used to explain the scene categorization advantage when scenes contained consistent vs. inconsistent objects). However, the fact that a relatively simple (V1-like) model of feature computation, seems sufficient to account for behavioral responses does not necessary imply that rapid scene categorization is based on low-level visual processing. We have tested alternative visual representations based on common features used in computer vision and found all these models to be relatively correlated. This could possibly reflect a limitation inherent to the ever limited size of natural image databases [55] as well as possible inherent biases such as photographers selecting vantage points [56]. Note that such image bias is quite different from the “natural bias” reported here in terms of differences in perceptual discriminability across categorization tasks, which is likely to reflect physical properties of our visual environment as opposed to biases in the image dataset per se.

In addition, while the superordinate advantage has been described for other classes of stimuli beyond scenes such as animals [16] or faces [5, 6], we have here only considered the relevance of the model for scene categorization. The use of a similar framework for other type of classifications would be likely to require more elaborated visual representations. In theory, it should be relatively straightforward to test additional perceptual representations—possibly reflecting higher level visual processes (see [23] for a review).

A possible neural correlate for decision boundaries includes neurons with category-like tuning found throughout the cortex such as within the ventral stream, the prefrontal cortex (PFC) and the parietal cortex [57] and/or attentional processes that would differentially modulate individual feature dimensions according to their task diagnosticity [58]. Perceptual spaces in practice tend to be more flexible than assumed in the model as novel features can be learned (i.e., the meaning of some of the dimensions may change and/or dimensions may be added as a result of learning and plasticity) and perceptual spaces can be reshaped by task history and other cognitive factors [59]. Alternative categorization algorithms to the proposed decision boundary have been described based on either the distance to category prototypes [60] or the distance to individual exemplars [61]. The proposed discriminability measures based on the distance between stimuli and decision boundaries could be easily extended to distances to exemplars or prototypes [62]. While it is expected that a better model of the categorization process should improve the fit to behavioral data, it is unlikely to change any of our conclusions, since categorization models tend to produce similar behavioral predictions.

Overall, our study provides a computational level explanation for systematic variations found in behavioral responses for rapid categorization tasks across taxonomic levels, challenging several existing theories of visual processing and suggesting, instead, that observed differences in behavioral responses may simply reflect natural variations in perceptual discriminability.

## Supporting Information

S1 Text Supplementary materials and methods including details on the comparison between different types of visual descriptors and classifiers. (PDF)

S1 File Supplementary file containing all image stimuli used and corresponding behavioral responses from human participants. (ZIP)

S1 Fig Sketch of the gist visual representation used. The response of a battery of filters at multiple orientations and spatial frequencies is first computed for an individual image. These filter responses are then spatially pooled to yield a 512-dimensional (gist) feature vector.
(TIF)

S2 Fig Correlation between visual representations. Simple visual representations like the gist tend to be relatively correlated with more complex ones including state-of-the-art visual descriptors from computer vision (see text for detail). This is true when correlating both the predicted class labels for individual train-test splits (A) and discriminability values computed across all train-test splits (B).
(TIF)
