# Supplementary material for: Explaining the Timing of Natural Scene Understanding with a Computational Model of Perceptual Categorization
Source: PLoS Comput Biol. 2015 Sep 3;11(9):e1004456. doi: 10.1371/journal.pcbi.1004456 (PMC4559373; doi:10.1371/journal.pcbi.1004456)
Supplement: S1 Text — (PDF) [file pcbi.1004456.s001.pdf]

Supplementary materials and methods

Explaining the timing of natural scene understanding with a computational model of perceptual categorization

Imri Sofer, Sébastien M. Crouzet, Thomas Serre

Cognitive, Linguistic & Psychological Sciences Department, Brown Institute for Brain Science, Brown University, Providence, RI, USA

Descriptors and classifiers comparison

We compared the "gist" visual representation (S1 Fig) used here to 4 popular visual descriptors, which achieved high accuracy in scene classification experiments (see [26] for details): dense SIFT, SSIM, HOG, and texton histograms. We used the general methodology as described in *Methods* using pre-computed features which are publicly available [26]. For this experiment, 80% of the images in the active set were used for training (1,440 images) and the remaining images used for testing. We first evaluated the classification agreement between the gist and other descriptors by computing the frequency at which the gist predicted the same image label as the other descriptors. We found that all descriptors tended to classify the images similarly to the gist, with average classification agreement of $r = 0.85$ ($p < 10^{-4}$; S2A Fig).

In addition, we also evaluated the correlation between the predicted discriminability values from different visual representations after controlling for class labels. This statistic captures the within-category correlation between two descriptors. Controlling for class labels was done by replacing the discriminability value of man-made images with 1 minus the discriminability value. Then the Pearson correlation coefficients between the gist and other descriptors were computed. We found that all descriptors tended to assign similar discriminability values compared to the gist descriptor, with average discriminability value correlation of $r = 0.47$ ($p < 10^{-4}$; S2B Fig). These correlations between visual representations most likely reflect low-level biases in the image dataset. Despite the fact that the dataset used is much larger that standard datasets used in vision science, it remains relatively small compared to the inherent size of the space of all natural scenes.

We further compared the robustness of the linear classifier used here (regularized logistic regression) to a more complex decision function (Support Vector Machine with radial basis function kernel, SVM-RBF). For this comparison, the scikit-learn module was used using the same training/test procedure for both classifiers. The predictions of the SVM-RBF were transformed to probabilities using the scikit-learn interface. Again, we found both the classification agreement between the two classifiers ($r = 0.89$; $p < 10^{-4}$) and their discriminability value correlation ($r = 0.73$; $p < 10^{-4}$) to be high.
