# Supplementary figures and images for: Explaining the Timing of Natural Scene Understanding with a Computational Model of Perceptual Categorization
Source: PLoS Comput Biol. 2015 Sep 3;11(9):e1004456. doi: 10.1371/journal.pcbi.1004456 (PMC4559373; doi:10.1371/journal.pcbi.1004456)

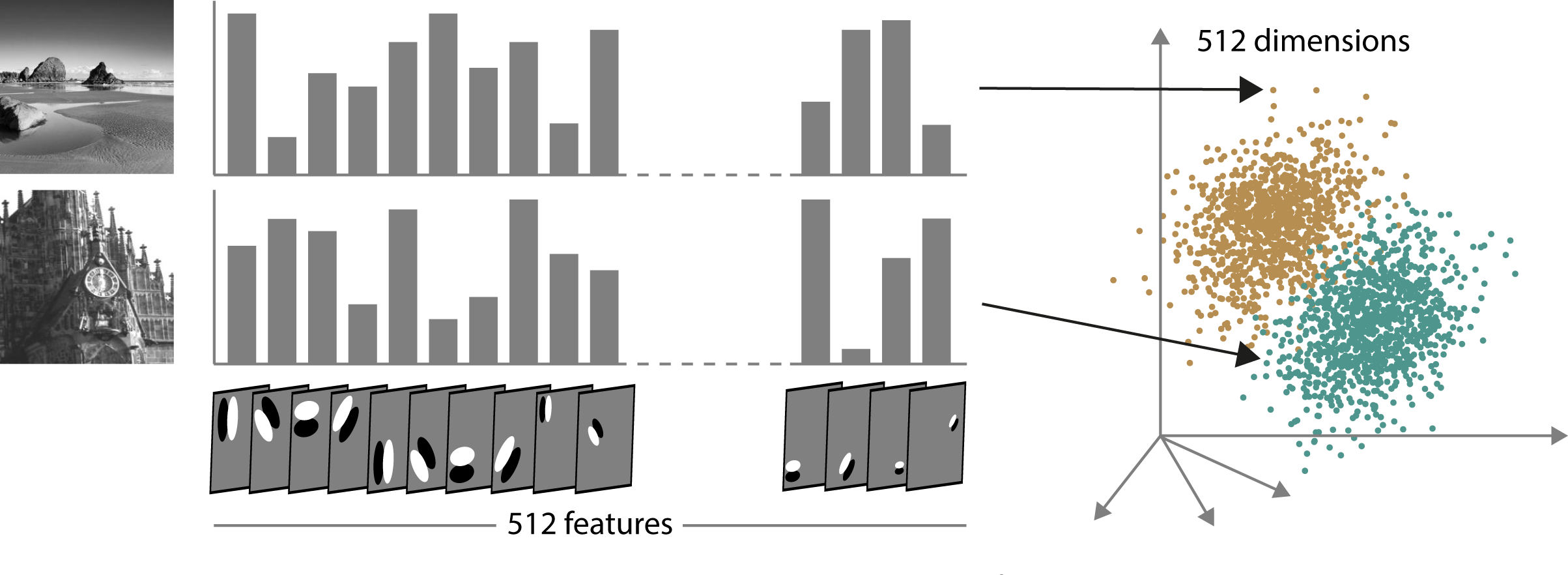

Supplement: S1 Fig — The response of a battery of filters at multiple orientations and spatial frequencies is first computed for an individual image. These filter responses are then spatially pooled to yield a 512-dimensional (gist) feature vector. (TIF) [file pcbi.1004456.s003.tif]

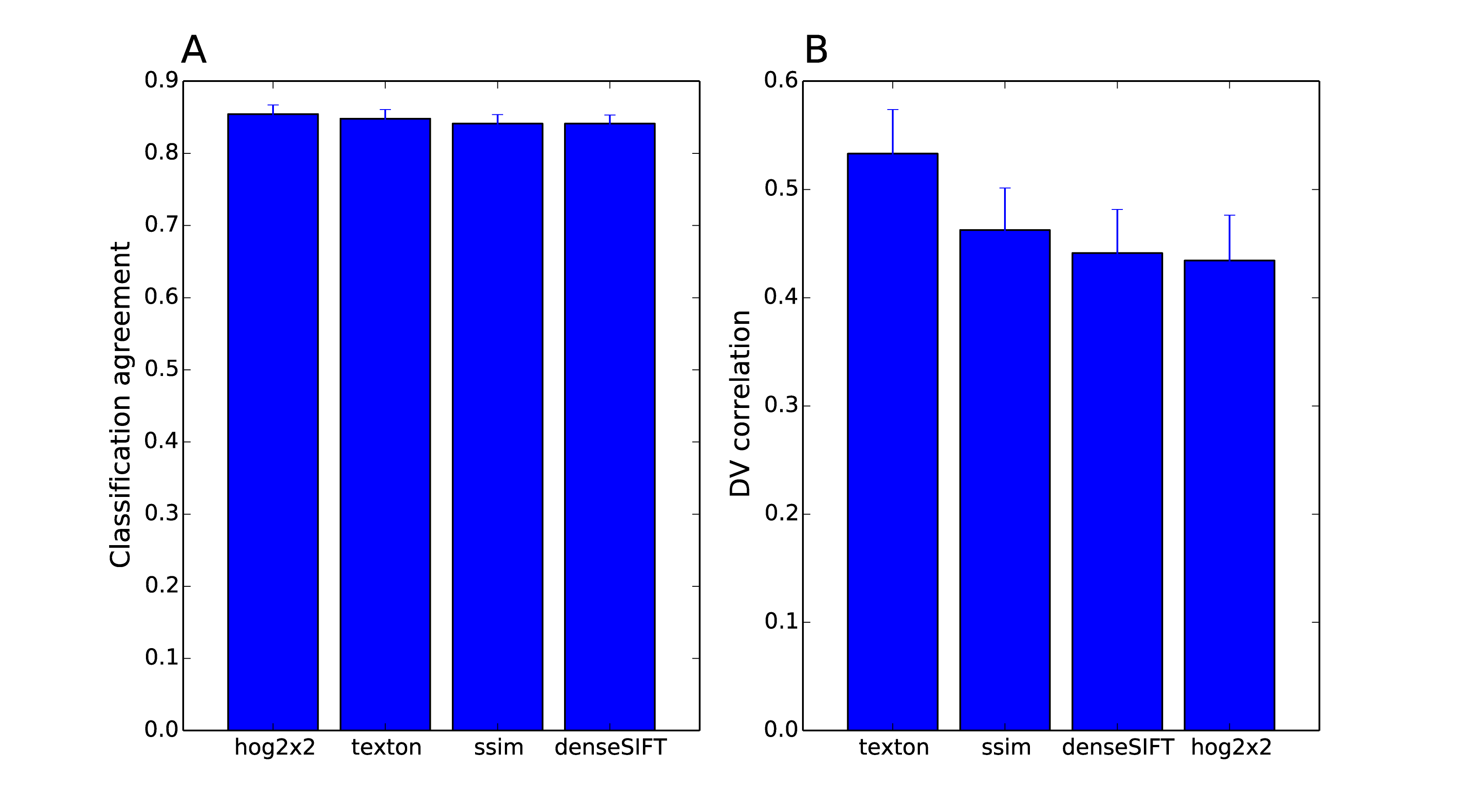

Supplement: S2 Fig — Simple visual representations like the gist tend to be relatively correlated with more complex ones including state-of-the-art visual descriptors from computer vision (see text for detail). This is true when correlating both the predicted class labels for individual train-test splits (A) and discriminability values computed across all train-test splits (B). (TIF) [file pcbi.1004456.s004.tif]
